# Inhibition of Intestinal Bile Acid Transporter Slc10a2 Improves Triglyceride Metabolism and Normalizes Elevated Plasma Glucose Levels in Mice

**DOI:** 10.1371/journal.pone.0037787

**Published:** 2012-05-25

**Authors:** Thomas Lundåsen, Eva-Marie Andersson, Michael Snaith, Helena Lindmark, Johanna Lundberg, Ann-Margret Östlund-Lindqvist, Bo Angelin, Mats Rudling

**Affiliations:** 1 Karolinska Institute, Department of Endocrinology, Metabolism and Diabetes, Department of Medicine, and Molecular Nutrition Unit, Center for Biosciences, NOVUM, Karolinska University Hospital Huddinge, Stockholm, Sweden; 2 Bioscience, AstraZeneca R&D, Mölndal, Sweden; 3 Lead Generation, AstraZeneca R&D, Mölndal, Sweden; 4 Transgenics and Comparative Genomics, at AstraZeneca R&D, Mölndal, Sweden; University of Michigan Medical School, United States of America

## Abstract

Interruption of the enterohepatic circulation of bile acids increases cholesterol catabolism, thereby stimulating hepatic cholesterol synthesis from acetate. We hypothesized that such treatment should lower the hepatic acetate pool which may alter triglyceride and glucose metabolism. We explored this using mice deficient of the ileal sodium-dependent BA transporter (Slc10a2) and ob/ob mice treated with a specific inhibitor of Slc10a2. Plasma TG levels were reduced in Slc10a2-deficient mice, and when challenged with a sucrose-rich diet, they displayed a reduced response in hepatic TG production as observed from the mRNA levels of several key enzymes in fatty acid synthesis. This effect was paralleled by a diminished induction of mature sterol regulatory element-binding protein 1c (Srebp1c). Unexpectedly, the SR-diet induced intestinal fibroblast growth factor (FGF) 15 mRNA and normalized bile acid synthesis in *Slc10a2−/−* mice. Pharmacologic inhibition of Slc10a2 in diabetic ob/ob mice reduced serum glucose, insulin and TGs, as well as hepatic mRNA levels of Srebp1c and its target genes. These responses are contrary to those reported following treatment of mice with a bile acid binding resin. Moreover, when key metabolic signal transduction pathways in the liver were investigated, those of Mek1/2 - Erk1/2 and Akt were blunted after treatment of ob/ob mice with the Slc10a2 inhibitor. It is concluded that abrogation of Slc10a2 reduces hepatic Srebp1c activity and serum TGs, and in the diabetic ob/ob model it also reduces glucose and insulin levels. Hence, targeting of Slc10a2 may be a promising strategy to treat hypertriglyceridemia and diabetes.

## Introduction

Bile acids (BAs) are crucial for the intestinal absorption of dietary fatty acids, cholesterol and fat-soluble vitamins. Disturbances in BA metabolism can initiate malabsorption and cholelithiasis but may also be important in the development of hyperlipidemia and arteriosclerosis [1]. Over 95% of the intestinal BAs are absorbed and returned to the liver. Since the entire pool of BAs circulates 4–10 times per day, a reduced intestinal absorption of BAs by merely a few percent will significantly increase the daily fecal excretion of BAs. The intestinal absorption of BAs is largely an active process where the BA transporter Slc10a2, also known as ileal sodium/BA cotransporter (Ibat) or apical sodium-dependent BA transporter (Asbt), is of major importance [2]. The BAs regulate their own synthesis, thereby securing a constant pool of BAs [1]. This is largely exerted at the level of the rate-limiting microsomal enzyme cholesterol 7α-hydroxylase (Cyp7a1) by feedback inhibition [1]. In addition to this direct feed-back by the BAs in the liver, mediated by the farnesoid X receptor (Fxr), regulation of Cyp7a1 gene expression is also exerted by cholesterol through an liver X receptor (Lxr)-mediated feed-forward mechanism in rodents [3] and by various hormones, although the mechanisms for the latter are still unclear. Finally, hepatic BA synthesis may also be controlled by fibroblast growth factor (FGF)15 (FGF19 in humans) secreted from the intestine. The production of this proposed enterokine is stimulated by activation of Fxr presumably when BAs traverse the enterocyte. Circulating FGF15 is thought to bind to its receptor FGFr4 in the liver leading to suppression of Cyp7a1 [1].

Interruption of the enterohepatic circulation of BAs can be achieved by the removal of the distal ileum [4], [5], or by the administration of BA-binding resins [6]. These treatments elicit a compensatory increase in BA synthesis which enhances the hepatic demand for cholesterol. This is met by an induced hepatic synthesis of cholesterol and an increased hepatic uptake of circulating cholesterol-rich lipoproteins; the latter leading to reduced plasma cholesterol levels [6], [7]. The effects of Slc10a2 deficiency on BA metabolism in mice have previously been described [2]. In male *Slc10a2−/−* mice, the fecal BA excretion is increased by 8 to 24-fold, and the total pool of BAs is reduced by ∼80%. No other major active uptake mechanism for intestinal BAs was identified. The activity of the rate-limiting enzyme in BA synthesis, Cyp7a1, was increased 2.5-fold and its mRNA levels and protein mass were increased 4-fold. The loss of BAs in *Slc10a2−/−* animals creates an increased hepatic demand for cholesterol that in turn consumes precursors for cholesterol synthesis. Since cholesterol and triglyceride (TG) synthesis share the mutual precursor acetyl-CoA, an important question is therefore whether chronic BA loss might alter hepatic TG and glucose homeostasis. In humans, treatment with BA-binding resins often increases plasma TGs due to enhanced VLDL synthesis [8], [9], [10], and BA malabsorption has been postulated as an underlying defect in familial hypertriglyceridemia [11].

In the present study, the aim was to evaluate if interruption of BA circulation by genetic deletion or pharmacological inhibition of Slc10a2 alters TG and glucose metabolism. Surprisingly, mice deficient in Slc10a2 had reduced plasma TG levels. Further, the increase in hepatic TG content following feeding a sucrose-enriched diet was attenuated in *Slc10a2−/−* mice. In line with this finding, expression of sterol regulatory element-binding protein 1c (Srebp1c) and its target genes were reduced in *Slc10a2−/−* mice. Specific pharmacological inhibition of the Slc10a2 protein in hyperlipidemic ob/ob mice reduced blood glucose, insulin and serum TGs. Also, hepatic Srebp1c lipogenic target genes and gluconeogenic genes were reduced, together with an altered signal transduction pattern. The data show that interruption of BA recirculation through abrogation of Slc10a2 reduces plasma TG levels accompanied by reduced hepatic Srebp1c, a response previously not recognized.

## Materials and Methods

### Ethics Statement

All animal care and experiments were conducted in accordance with accepted national standards of humane animal care and approved by the Ethics Committee of the University of Gothenburg (Permit Numbers: 140–2005 and 84–2002).

### Animals and Diets

**Figure 1:**
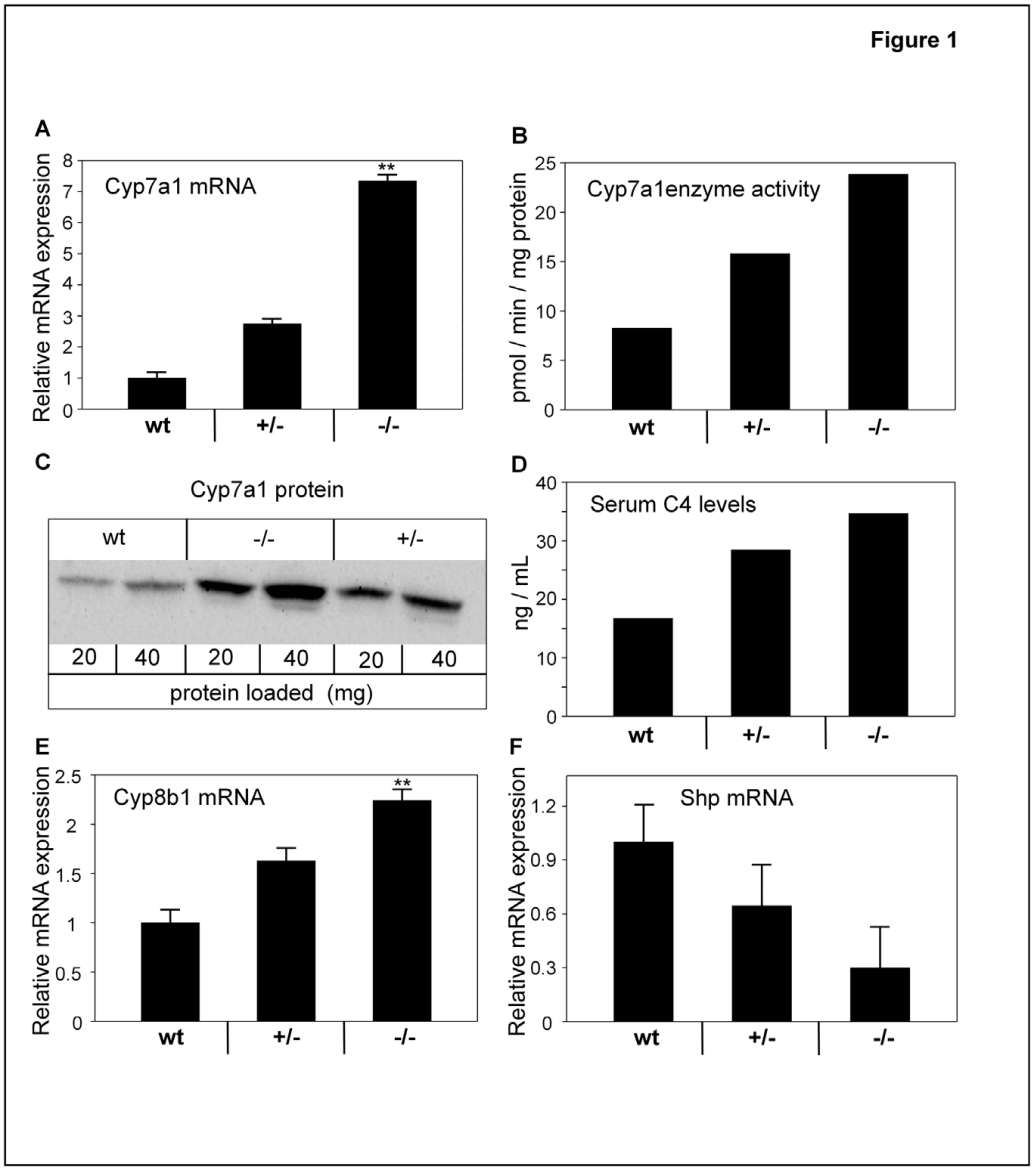
Disruption of the ileal BA transporter gene Slc10a2 **activates enzymes in BA synthesis.** (A) mRNA levels of cholesterol 7α-hydroxylase (Cyp7a1) in livers of wt, *Slc10a2+/−* and *Slc10a2−/−* animals. (B) Cyp7a1 enzymatic activity in pooled microsomal samples from livers of wt, *Slc10a2+/−* and *Slc10a2−/−* animals. Results shown are mean of two analyses. (C) Protein levels of Cyp7a1 in livers of wt, *Slc10a2+/−* and *Slc10a2−/−* mice determined in pooled microsomal samples by immunoblot using a Cyp7a1-specific antibody. 20 µg and 40 µg of protein were used per sample, respectively. The results are representative of three separate immunoblots. Note the loading order of the samples. (**D**) Serum levels of the Cyp7a1 activity marker 7α-hydroxy-4-cholesten-3-one (C4) were assayed as an indirect measure of Cyp7a1 enzymatic activity in pooled serum samples of wt, *Slc10a2+/−* and *Slc10a2−/−* animals. Data show mean of two separate measurements. (**E**) hepatic mRNA levels of 12α-hydroxylase (Cyp8b1), and (**F**) hepatic small heterodimer partner (Shp) mRNA levels of wt, *Slc10a2+/−* and *Slc10a2−/−* mice. mRNA levels for the wt mice were normalized to 1. Data are expressed as mean ± standard error (SEM) for the mRNA analysis. wt littermates (n = 10), *Slc10a2+/−* (n = 9) and *Slc10a2−/−* (n = 10) for A-F. Significances of differences between groups was tested by Dunnetts test; a p-value < <0.01 is denoted **.

**Figure 2:**
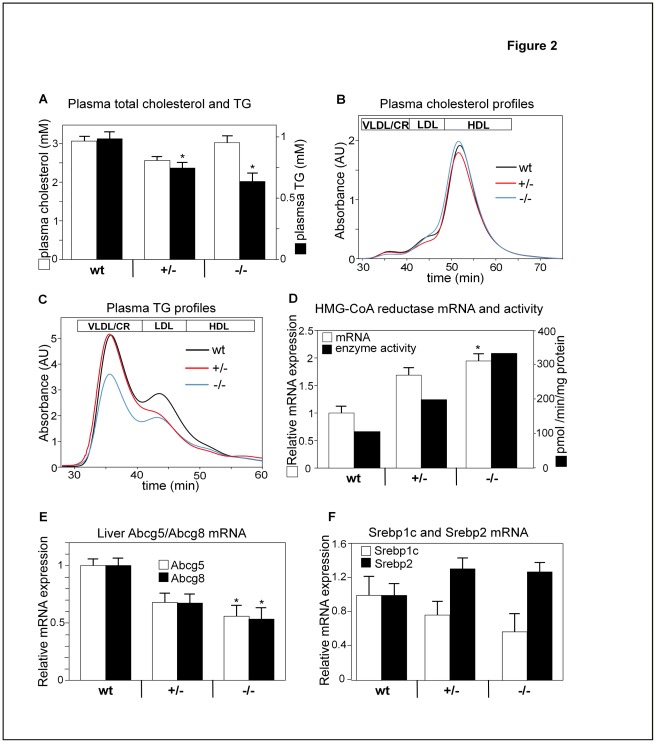
Ablation of the Slc10a2 gene lowers the levels of plasma s TGs but not cholesterol. (A) Total plasma cholesterol and TGs from wt, *Slc10a2+/−* and *Slc10a2−/−* mice. (B) Plasma profiles of cholesterol and TGs (C) were analysed from wt, *Slc10a2+/−* and *Slc10a2−/−* animals by fast protein liquid chromatography (FPLC). Lines represent means of all individuals from each group, respectively. Lipoprotein fractions are indicated, n = 9–10. (D) Hepatic 3-hydroxy-3-methyl-glutaryl-CoA (HMG-CoA) reductase mRNA levels and enzymatic activity of wt, *Slc10a2+/−* and *Slc10a2−/−* mice. HMG-CoA reductase enzymatic activity was analyzed from pooled hepatic microsomal samples and represents mean of two measurements. (n = 9–10). (E) Hepatic mRNA levels of the sterol transporters ATP-binding cassette sub-family G member 5 (Abcg5) and Abcg8 in wt, *Slc10a2+/−* and *Slc10a2−/−* mice. (F) Hepatic mRNA levels of sterol regulatory element-binding protein 1c (Srebp1c) and Srebp2, in wt, *Slc10a2+/−* and *Slc10a2−/−* mice. mRNA levels in wt mice were normalized to 1. Data are expressed as mean ± standard error (SEM) for the mRNA and total plasma cholesterol and TGs analyzes. wt littermates (n = 10), *Slc10a2*+/− (n = 9) and *Slc10a2*−/− (n = 10) for A-F. Significances of differences between groups was tested by Dunnetts test, a p-value <0.05 is denoted *.

**Figure 3:**
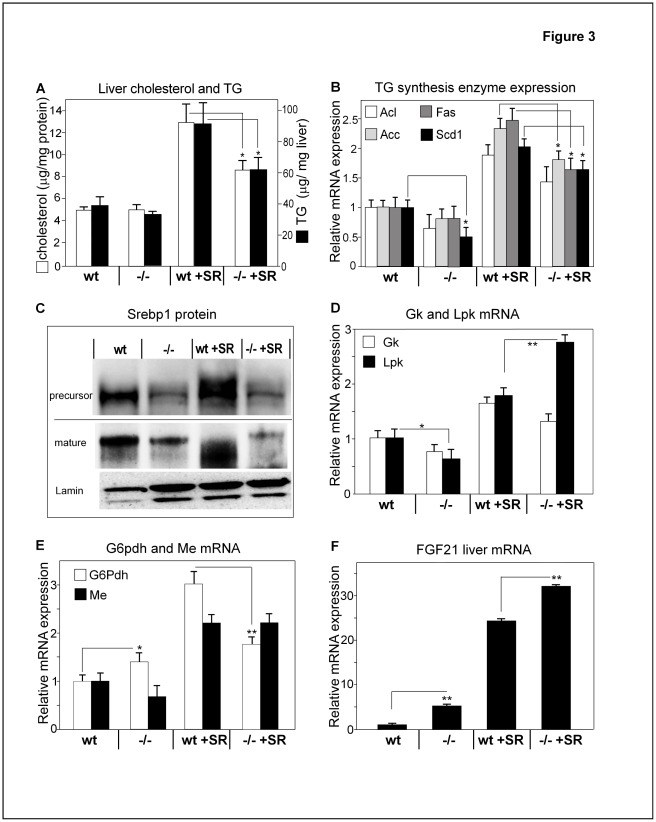
*Slc10a2*−/− mice display lower hepatic TGs and cholesterol than wild type mice. (A) Liver TGs and cholesterol content were analyzed from a total of four groups; wt and *Slc10a2−/−* mice fed either standard mouse chow or a sucrose-rich (SR) diet (Materials and Methods) for two weeks. (B) Hepatic mRNA levels of ATP-citrate lyase (Acl), acetyl-CoA carboxylase (Acc), fatty acid synthase (Fas) and stearoyl-CoA desaturase 1 (Scd1), enzymes involved in fatty acid synthesis from wt or *Slc10a2−/−* animals, as in (A). (C) Sterol regulatory element-binding protein 1 (Srebp1) immunoblots were performed on pooled liver cytoplasmic and nuclear protein preparations, respectively, from wt or *Slc10a2−/−* mice fed a regular chow or a SR diet using an antibody specific for the N’-terminus of Srebp1. An antibody against lamin was used as nuclear loading control. The blot is representative of three separate immunoblots. Hepatic mRNA expression levels of (D), glucokinase (Gk); pyruvate kinase (Lpk); (E) glucose-6-phosphate dehydrogenase (G6pdh); malic enzyme (Me). mRNA values in the wt group fed regular chow were normalized to 1. (F) Fibroblast growth factor 21, FGF(21). mRNA values in the wt group on regular chow were normalized to 1. Data are expressed as mean ± standard error (SEM) for the mRNA, hepatic cholesterol and TG analysis. wt (n = 6), *Slc10a2−/−* (n = 5), wt + SR (n = 6) and *Slc10a2+/−* +SR (n = 6) for A-D. Significances of differences between groups were tested by Student’s t test, a p-value <0.05 is denoted *. p<0.01 is denoted **.

*Slc10a2+/−* and *Slc10a2−/−* mice were generated at AstraZeneca R&D, Mölndal, as detailed in supplemental experimental procedures (Text S1) based on the outline of Dawson et al [2]. Throughout the study male mice were used. In the experiments shown in Figure 1 and 2 mice were 20 weeks and in Figure 3, 33 weeks old. A sucrose-rich diet (61% sucrose, D12329, Research Diets, NJ), together with drinking water supplemented with 10% fructose, was used for the high-carbohydrate experiment. Animals had free access to the diet for two weeks. If not stated otherwise, all other animals received standard mouse chow (R3; Lactamin AB, Stockholm, Sweden) containing (energy %) 12% fat, 62% carbohydrates, and 26% protein, with a total energy content of 12.6 kJ/g and tap water. There were no additions of cholesterol to the diets. Male ob/ob animals 10–12 weeks old were from Taconic A/S, DK. Ob/ob animals were gavaged with a specific Slc10a2 inhibitor AZD 7806 (AstraZeneca R&D, Mölndal, Sweden) that is not absorbed and therefore lacks systemic side effects, (AZD7806 is not equivalent to PR835 in ref. [12]) or a control vehicle for 11 days. Animals had free access to food and water. Since it is known that at least BA and cholesterol synthesis pathways are subject to diurnal variation, including higher order circadian regulation, blood and tissue sampling were standardized and performed in the morning to minimize circadian effects. All animal care and experiments were conducted in accordance with accepted standards of humane animal care and approved by the Ethics Committee of the University of Gothenburg (Permit Numbers: 140–2005 and 84–2002).

### Plasma Analysis

Blood was centrifuged, and plasma was analyzed for total cholesterol and TGs using the IL Tests cholesterol 181618–10 and TG 181610–60 kits on the Monarch 2000 system (IL Scandinavia, Gothenburg, Sweden). Lipoprotein cholesterol profiles were obtained by separation of 10 µl of plasma using a micro fast protein liquid chromatography (FPLC) system [13]. Plasma insulin levels were analyzed using a rodent insulin RIA kit (Linco, St. Charles, MI). Total plasma glucose was analyzed using the IL Test (IL Scandinavia, Gothenburg, Sweden) on the Monark 2000 system. Blood glucose was determined using a Bayer Elite glucometer (Bayer diagnostics, Germany). Plasma free fatty acids were analyzed employing a commercial NEFA kit (Wako Chemicals USA Inc. Richmond, Virginia). Serum levels of 7α-hydroxy-4-cholesten-3-one (C4) were used as an indirect measurement of Cyp7a1 activity, and analyzed in either pooled or individual plasma samples by high pressure liquid chromatography [14].

### Enzymatic Activities

Enzyme activities of 3-hydroxy-3-methylglutaryl-CoA (HMG-CoA) reductase and of cholesterol 7alpha hydroxylase (Cyp7a1) were assayed in hepatic microsomes, as described [15]. In this assay there was no addition of cholesterol - the endogenous cholesterol is utilized as substrate.

### RNA Extraction and Quantitative Real Time PCR

Total RNA was extracted from frozen livers or distal ileum with TRIzol reagent (Invitrogen, Carlsbad, CA). The RNA was DNase-treated with RQ1 Dnase (Promega, Madison, WI). cDNA synthesis and quantitative real time PCR was performed employing HPRT as endogenous control [16]. Primer and probe information are available on request.

### Immunoblotting of Liver Proteins

A ligand blot using ^125^I-labeled rabbit β-VLDL as probe was used to detect LDL receptors in liver as described [17].

Hepatic scavenger receptor class B type 1 (Srb1) was assayed by immunoblot using a rodent specific rabbit polyclonal antibody (Novus Biologicals Inc., Littleton, CO) as described [18]. Cyp7a1 protein levels were assayed in liver microsomal protein samples by immunoblot using a rabbit polyclonal antibody directed against the C’-terminus of the Cyp7a1 [16].

To detect Srebp1 protein, cytoplasmic and nuclear protein preparations from liver were performed using the NE-PER reagent (Pierce), including Complete protease inhibitor (Roche), 1 mM phenyl-methylsulfonyl fluoride, 0.5 mM leupeptin, 5 µg/ml Calpain inhibitor I (Biomol, PA), following the manufacturer’s instructions. Cytoplasmic and nuclear liver protein fractions, 50 µg and 25 µg respectively, were electrophoresed on NuPage Bis-Tris gels (Invitrogen) and transferred to nitrocellulose membranes. Membranes were blocked in 5% skim milk powder and incubated with a mouse monoclonal antibody raised against the N’-terminus of Srebp1 (Labvision Corporation, CA) at 1.5 µg/mL in 5% skim milk powder for two hours at room temperature. An HRP conjugated goat anti-mouse F(ab’)_2_ antibody (Pierce) was used for detection of specific signals together with Supersignal reagent (Pierce) and a Fuji BAS 1800 analyzer (Fuji Photo Film Co.). Lamin AB was obtained from Cell signaling Technology®, Boston, Mass.

To analyze phosphorylated liver proteins by Western blot, total liver protein homogenates were prepared from frozen tissue by homogenization using a polytron followed by sonication in a buffer containing 20 mM Tris-Hcl, pH 7,4, 1% Triton X-100, 10% glycerol, 150 mM NaCl, 2 mM EDTA, 25 mM beta-glycerophosphate, 20 mM sodium floride, 1 mM sodium orthovanadate, 2 mM sodium pyrophosphate, 1 mM benzamidine, 1 mM phenyl-methylsulfonyl fluoride, 0.5 mM leupeptin, Complete protease inhibitor (Roche), and then centrifuged at 14 000 rpm in a microcentrifuge, and the supernatant was recovered. 50 µg of protein was loaded on NuPage Bis-Tris gels (Invitrogen) and transferred to nitrocellulose membranes. Membranes were blocked in Starting Block-PBS (Pierce) and incubated in 3% BSA with phosphorylation site specific antibodies, pAkt1, pErk1/2 and pMek1/2, as indicated in figures, overnight at +4°C, then stripped with Restore (Pierce), reprobed with antibodies detecting total amounts of Akt1, Mek1/2 and Erk1/2, and finally stripped and reprobed with an antibody against beta-actin (Abcam) to verify equal gel loading. Antibodies against kinases were purchased from Cell Signaling Technology, Inc. Blots were developed as described above.

### Determination of liver TGs and cholesterol

Liver cholesterol and TG were extracted as previously described [19] and determined using commercially available kits (Roche Applied Science, Indianapolis).

### Statistics

Data are shown as mean +/− SEM and were log transformed prior to statistical analysis. The significance of differences between groups was tested as indicated in Figure legends, by 1-way ANOVA followed by post-hoc comparisons according to Dunnett or by Student’s t-test using GraphPad Prism® Software.

## Results

### Generation of *Slc10a2−/−* Mice

Generation of *Slc10a2−/−* mice was performed as described in detail in supplemental experimental procedures (Text S1). Southern blotting, quantitative real time PCR and immunoblotting confirmed appropriate targeting and lack of Slc10a2 expression in the null mice (Figure S1 and not shown.) *Slc10a2−/−* animals were viable and fertile. No abnormalities in behaviour, gross appearance, body weight or survival were seen, consistent with the previous report by Dawson et al. [2].

### Increased BA synthesis in *Slc10a2+/−* and *Slc10a2−/−* Mice

An established response following disruption of the enterohepatic circulation of BAs is an induced synthesis of BAs [2], [4], [5], [6], [20]. Indeed, when the hepatic mRNA levels for Cyp7a1, the rate-limiting enzyme in BA synthesis, were assayed by quantitative real time PCR (qrtPCR), there was an ∼7-fold induction in *Slc10a2−/−* mice (Figure 1A). Also, in the *Slc10a2+/−* mice there was a clear but less pronounced (∼3-fold) increase in Cyp7a1 mRNA.

Consistent with the Cyp7a1 mRNA increase, the enzymatic activity and the protein mass of Cyp7a1 were higher in both *Slc10a2+/−* and *−/−* mice, when examined in pooled microsomes (Figures 1B and 1C). The Cyp7a1 reaction product 7α-hydroxy-4-cholesten-3-one (C4), present in blood serum, has been demonstrated to be an accurate marker of Cyp7a1 enzyme activity [14]. Analysis of pooled serum from both heterozygous and homozygous animals revealed higher serum C4 levels as compared to control animals (Figure 1D).

In conjunction with the observed increase in Cyp7a1, the mRNA levels for the sterol 12α-hydroxylase (Cyp8b1) were also induced dose-dependently (Figure 1E). When the circulation of BAs is disrupted, the amount of available ligand for the hepatic nuclear BA receptor Fxr decreases. In line with this, decreased hepatic mRNA levels of the Fxr target gene small heterodimer partner (Shp), a suppressor of Cyp7a1 gene transcription, were found in heterozygous and homozygous mice, with the most pronounced change in the latter group (Figure 1F).

### Lowered Plasma TGs in *Slc10a2+/−* and *Slc10a2−/−* Mice

Plasma total TGs were significantly reduced by 22% in *Slc10a2+/−* mice and by 35% in *Slc10a2−/−* mice compared to controls (Figure 2A). The plasma cholesterol and TG lipoprotein profiles were then analyzed by FPLC (Figure 2B and C). The plasma cholesterol profiles did not vary notably between controls and homozygous animals. However, larger changes were found in the plasma TG profiles of these mice (Figure 2C). In *Slc10a2+/−* mice, TGs were reduced within LDL, whereas in *Slc10a2−/−* mice TGs were reduced in both VLDL- and LDL (Figure 2C). Plasma glucose and insulin were not altered in *Slc10a2−/−* animals (not shown).

### Adaptation of Hepatic Cholesterol Metabolism to BA Deficiency

There were no changes in the hepatic expression of the LDL-receptor or the HDL-receptor (Srb1), neither at mRNA nor at protein levels (not shown). The high demand for cholesterol as substrate for Cyp7a1 in the livers of *Slc10a2+/−* and *Slc10a2−/−* mice was reflected in increased enzymatic activity of the hepatic HMG-CoA reductase, with a 2-fold increase in the heterozygous and a 3.5-fold increase in homozygous animals, based upon analysis of pooled microsomal samples (Figure 2D). The increases in hepatic HMG-CoA reductase mRNA levels had a similar pattern, although smaller differences were observed between groups (Figure 2D). The gene expression of the hepatic sterol transporters ATP-binding cassette sub-family G member 5 (Abcg5) and Abcg8 were suppressed by up to 50% in a gene-dose dependent manner (Figure 2E).

In line with the findings for HMG-CoA reductase, the hepatic levels of Srebp2 mRNA were increased in both *Slc10a2+/−* and *Slc10a2*−/− mice (Figure 2F). Intriguingly, the regulation of the Srebp1c gene in the liver displayed an opposite pattern, with reduced mRNA levels in heterozygous and homozygous mice compared to controls, again in a gene-dose dependent manner (Figure 2F).

### Hepatic TG Production is Suppressed in *Slc10a2−/−* Mice

To further explore TG metabolism in this animal model of BA malabsorption, *Slc10a2−/−* and wt control animals received a sucrose-rich diet (SR diet) for two weeks to increase substrate availability, while animals on chow served as controls. For both diets, neither plasma glucose, insulin nor food intake were significantly different between *Slc10a2−/−* and respective wt control group, (not shown).

The hepatic TG content tended to be lower in *Slc10a2−/−* mice fed regular chow (Figure 3A). This difference was more evident on the SR diet where hepatic TGs increased by 120% in wt animals, whereas in *Slc10a2−/−* animals this increase was significantly blunted (Figure 3A). Also, hepatic cholesterol was lower in *Slc10a2−/−* mice fed the SR diet compared to wt controls on this diet (Figure 3A). Since the lower hepatic TG levels in *Slc10a2−/−* mice on the SR-diet could be due to decreased fatty acid synthesis we measured the mRNA levels of ATP-citrate lysase (Acl), acetyl-CoA carboxylase (Acc), fatty acid synthase (Fas) and stearoyl-CoA desaturase 1 (Scd1), by qRT PCR. The gene expression of these enzymes appeared reduced in the livers of *Slc10a2−/−* animals (Figure 3B); this finding was more pronounced when animals were challenged with the SR diet. The transcription factor Srebp1c is crucial for optimal activation of most genes in fatty acid synthesis [21]. The expression of the Srebp1c protein (mature and precursor form) was reduced in *Slc10a2−/−* mice, as evaluated by Western blot on cytoplasmic and nuclear proteins using an antibody against the N’-terminus of Srebp1c (Figure 3C).

Disruption of BA circulation by Slc10a2 gene deletion alters the expression of genes involved in hepatic glucose handling.

Hepatic TG synthesis is dependent on substrates in the glycolytic pathway and thus on the activity and gene expression of glucose metabolizing enzymes [22]. The mRNA levels of the hepatic glycolytic enzyme glucokinase (Gk) were unaltered in *Slc10a2−/−* animals as compared to wt controls, both on chow and on the SR diet (Figure 3D). However, the mRNA levels of liver pyruvate kinase (Lpk) were reduced 30% in *Slc10a2−/−* on chow (Figure 3D). mice Interestingly, feeding the SR diet resulted in a much stronger up-regulation of Lpk mRNA in *Slc10a2−/−* mice (4.3-fold), whereas in wt control animals there was a more modest 1.8-fold stimulation under the same conditions. Determination of the NADPH generating enzyme glucose-6-phosphate dehydrogenase (G6pdh) mRNA showed increased levels in *Slc10a2−/−* animals as compared to wt mice on regular chow (Figure 3E). Feeding the SR diet resulted in a three-fold increase of G6pdh mRNA in wt mice, whereas in *Slc10a2−/−* mice there was only a modest 60% increase (Figure 3E). It was therefore of interest to determine if the gene expression of malic enzyme (Me), also part of a NADPH-generating step converting malate to pyruvate, was changed in *Slc10a2−/−* livers. As seen from Figure 3E, Me mRNA tended to decrease in *Slc10a2−/−* mice on regular chow and increased during the SR diet to similar extents in wt and *Slc10a2−/−* animals.

### Normalization of Increased BA Synthesis in *Slc10a2*−/− Mice Following a SR Diet

**Figure 4:**
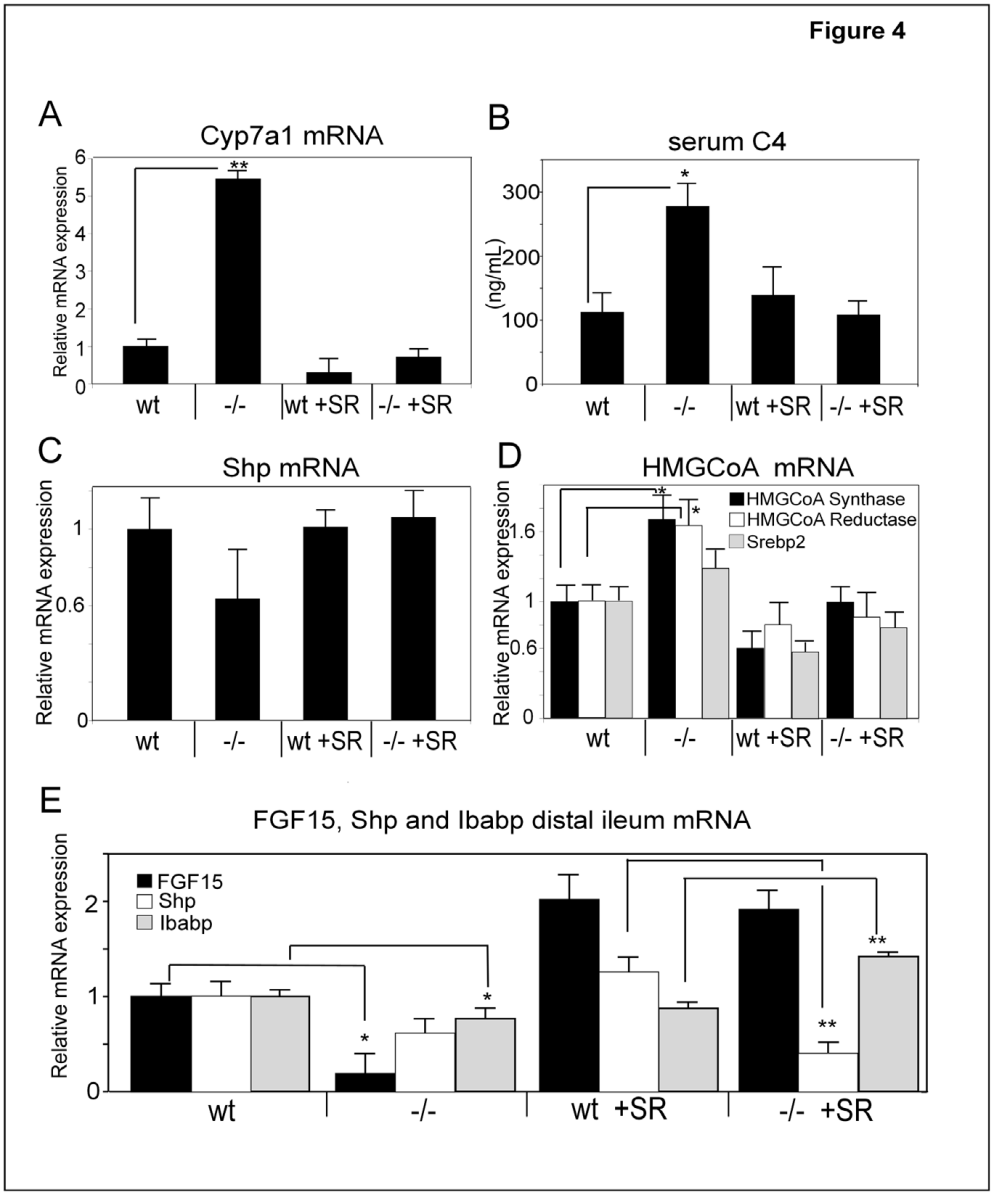
A sucrose-rich (SR) diet normalizes elevated CYP7A1 levels and induces the expression of the metabolic regulators FGF15 and FGF21 in *Slc10a2*−/− mice. Livers from wt and *Slc10a2*−/− mice fed regular chow or a SR diet were analysed for A), mRNA levels of Cyp7a1; B), Serum C4 levels, a marker of Cyp7a1 enzymatic activity; C), hepatic mRNA levels of SHP; and for D), HMGCoA synthase, HMGCoA reductase and SREBP2 mRNA. In panel E, FGF15, SHP and IBABP mRNA levels were determined in samples from distal ileum of wt and *Slc10a2*−/− mice fed regular diet or the SR diet. Data are expressed as mean ± standard error (SEM), n = 5–6. mRNA expression for the wt group on regular chow is normalized to 1. Significances of differences between groups were tested by Student’s t test, a p-value <0.05 is denoted *. p<0.01 is denoted **.

We next examined whether the SR diet had any effect on BA synthesis. Surprisingly, the elevated Cyp7a1 expression in *Slc10a2*−/− mice was dramatically suppressed to levels seen in wt animals when *Slc10a2*−/− mice were fed the SR diet (Figure 4A), and there was no difference in BA synthesis between *Slc10a2*−/− and wt on the SR diet. Also, the wt controls receiving the SR diet displayed a lower gene expression of Cyp7a1. Estimation of the level of BA synthesis by measurement of the serum marker C4 confirmed that the high enzymatic activity of Cyp7a1 was reduced following the SR diet to the *Slc10a2*−/− animals. However, the serum C4 levels were not reduced in wt animals on the SR diet (Figure 4B).

Since the stimulated Cyp7a1 expression in *Slc10a2*−/− appeared concomitantly with reduced SHP levels (Figure 1) we evaluated whether the suppressed Cyp7a1 expression may be linked to increased SHP levels. Assay of SHP mRNA (Figure 4C) showed that the reduced levels of SHP in the *Slc10a2*−/− mice were indeed normalized following the SR diet, although the reduction of SHP seen in the *Slc10a2*−/− mice in this experiment did not reach statistical significance.

We also examined the mRNA levels of key enzymes in sterol synthesis, HMGCoA synthase and HMGCoA reductase, during SR feeding since the increased demand for cholesterol in *Slc10a2*−/− mice was obviously met by increased de novo synthesis. The HMGCoA synthase and HMGCoA reductase mRNA levels were increased in *Slc10a2*−/− animals on normal diet whereas the levels were reduced in both wt and *Slc10a2*−/− mice when fed the SR diet (Figure 4D). These findings were in line with concomitantly altered mRNA levels for SREBP2 (Figure 4D). There was no difference in total liver cholesterol in *Slc10a2*−/− mice on chow as compared to wt mice (Figure 3A). However, the SR diet clearly increased total liver cholesterol in both types of mice - notably to a higher extent in the wt mice (Figure 3A).

### Inhibition of Slc10a2 in ob/ob Mice Reduces Plasma Glucose and TGs

**Figure 5:**
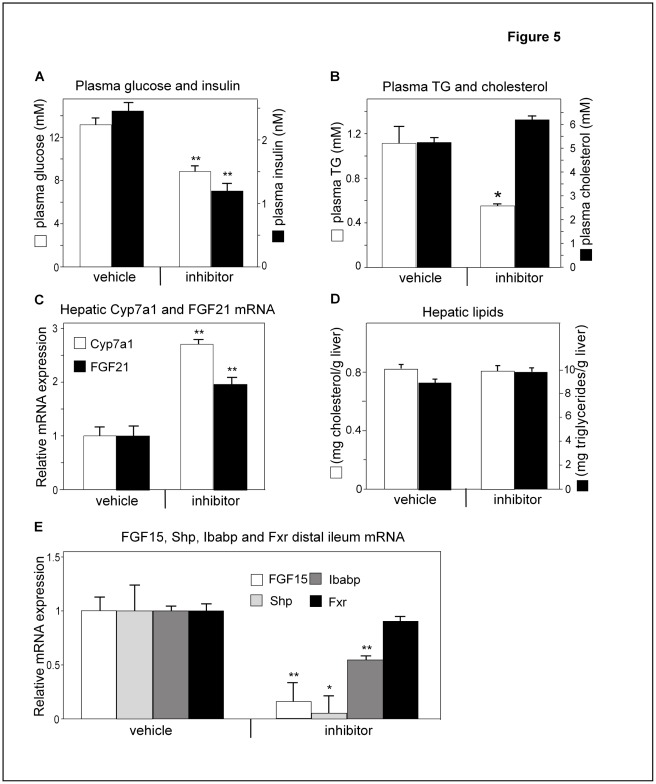
Inhibition of the Slc10a2 protein improves plasma glucose and TGs in ob/ob mice. Ob/ob mice were treated with a specific Slc10a2 protein inhibitor, (AZD7806), or control vehicle (vehicle) (see experimental procedures). (**A**) fasting levels of blood glucose and plasma insulin. (**B**) plasma total TGs and cholesterol. (**C**) Hepatic mRNA levels of fibroblast growth factor 21 (FGF21) and cholesterol 7α-hydroxylase (Cyp7a1). (**D**) Liver cholesterol and TGs. (**E**) Distal ileum mRNA levels of FGF15, small heterodimer partner (Shp), Ileal BA binding protein (Ibabp) and farnesoid X receptor (Fxr) from ob/ob animals treated with a Slc10a2 protein inhibitor. mRNA levels of the control vehicle treated animals are normalized to 1. Data are represented as mean ± standard error (SEM). Significances of differences between groups was tested by Student’s t test, a p-value <0.05 is denoted *. P<0.01 is denoted **.

Although serum glucose in *Slc10a2−/−* mice was not changed, these animals were hypolipidemic. We therefore evaluated if beneficial effects could be obtained in ob/ob mice, a model where plasma glucose and TGs are chronically elevated. Ob/ob mice were treated with the specific Slc2a10 inhibitor AZD7806 for 11 days. Fasting glucose levels were reduced 30% in drug-treated ob/ob mice compared to vehicle-treated animals (Figure 5A). Likewise, this treatment reduced fasting insulin levels by 50% and total plasma TG levels by 73%, whereas total plasma cholesterol tended to increase slightly (+16%) (Figure 5B).

As expected, the hepatic mRNA level of Cyp7a1 increased in drug-treated mice (Figure 5C), further, the level of HMG-CoA reductase increased 2-fold (not shown). When the hepatic contents for cholesterol and TGs were analyzed, there were no differences between inhibitor-treated and control animals (Figure 5D). To get indications of that Slc10a2 activity was blocked, the mRNA levels of the Fxr target genes FGF15, Shp and ileal BA binding protein (Ibabp) were assayed in samples from the distal ileum; they were all significantly reduced in response to treatment as could be expected from a diminished influx of BAs into the distal ileum (Figure 5E). The gene expression of Fxr was unaltered in the distal ileum following treatment with the Slc10a2 inhibitor (Figure 5E).

### Inhibition of Ileal Slc10a2 in ob/ob Mice Reduces Srebp1c and its Target Genes in the Liver

**Figure 6:**
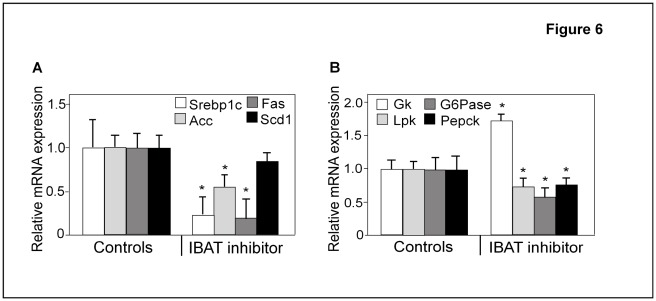
Inhibition of Slc10a2 reduces mRNA levels of enzymes in fatty acid and glucose metabolism. Ob/ob mice were treated with the specific Slc10a2 protein inhibitor, AZD7806 or control vehicle (Controls) (see experimental procedures). (**A**) Hepatic mRNA levels of Srebp1c, and its target genes acetyl-CoA carboxylase (Acc), fatty acid synthase (Fas) and stearoyl-CoA desaturase 1 (Scd1). (**B**) Hepatic mRNA levels of glucokinase (Gk), pyruvate kinase (Lpk), glucose 6-phosphatase (G6Pase) and phosphoenolpyruvate carboxykinase (Pepck) mRNA levels of the control vehicle treated animals are normalized to 1. Data are represented as mean ± standard error (SEM). Significances of differences between groups was tested by Student’s t test, a p-value <0.05 is denoted *.

Since dysregulation of Srebp1c has been reported to be a major determinant of the increased lipogenic response in ob/ob liver, ultimately leading to reduced hepatic insulin sensitivity, we next analyzed the gene expression of hepatic Srebp1c and three of its target genes. In accordance with the reduced Srebp1c levels observed in *Slc10a2−/−* mice both under basal and SR diet challenge, markedly reduced mRNA levels of Srebp1c were observed in response to administration of the Slc10a2 inhibitor to ob/ob mice (Figure 6A). Moreover, the mRNA levels of the Srebp1c liver target genes Acc and Fas were strongly decreased, by 50% and 80%, respectively (Figure 6A). However, the Scd1 mRNA level was not significantly altered compared to vehicle-treated controls (Figure 6A).

### Altered Expression of Hepatic Glucose Metabolic Genes in Slc10a2-inhibitor Treated ob/ob Mice

Next we investigated the expression levels of enzymes important for glucose handling in the livers of ob/ob mice treated with Slc10a2 inhibitor. As seen in Figure 6B, the mRNA level for Gk, the initial step in glycolysis, was increased in treated mice, in line with decreased blood glucose levels. However, in contrast, mRNA for the glycolytic gene Lpk was reduced by 30% (Figure 6B). A similar result for the Lpk mRNA was also found in the *Slc10a2−/−* livers (Figure 3D). We also analyzed the expression of the gluconeogenic genes glucose 6-phosphatase (G6Pase) and phosphoenolpyruvate carboxykinase (Pepck) (Figure 6B) and found that they were both reduced in the inhibitor-treated mice, in line with the finding of reduced blood glucose levels in these animals.

### Inhibition of Slc10a2 Reduces Akt and Mek1/2 Activity in ob/ob Mouse Liver

**Figure 7:**
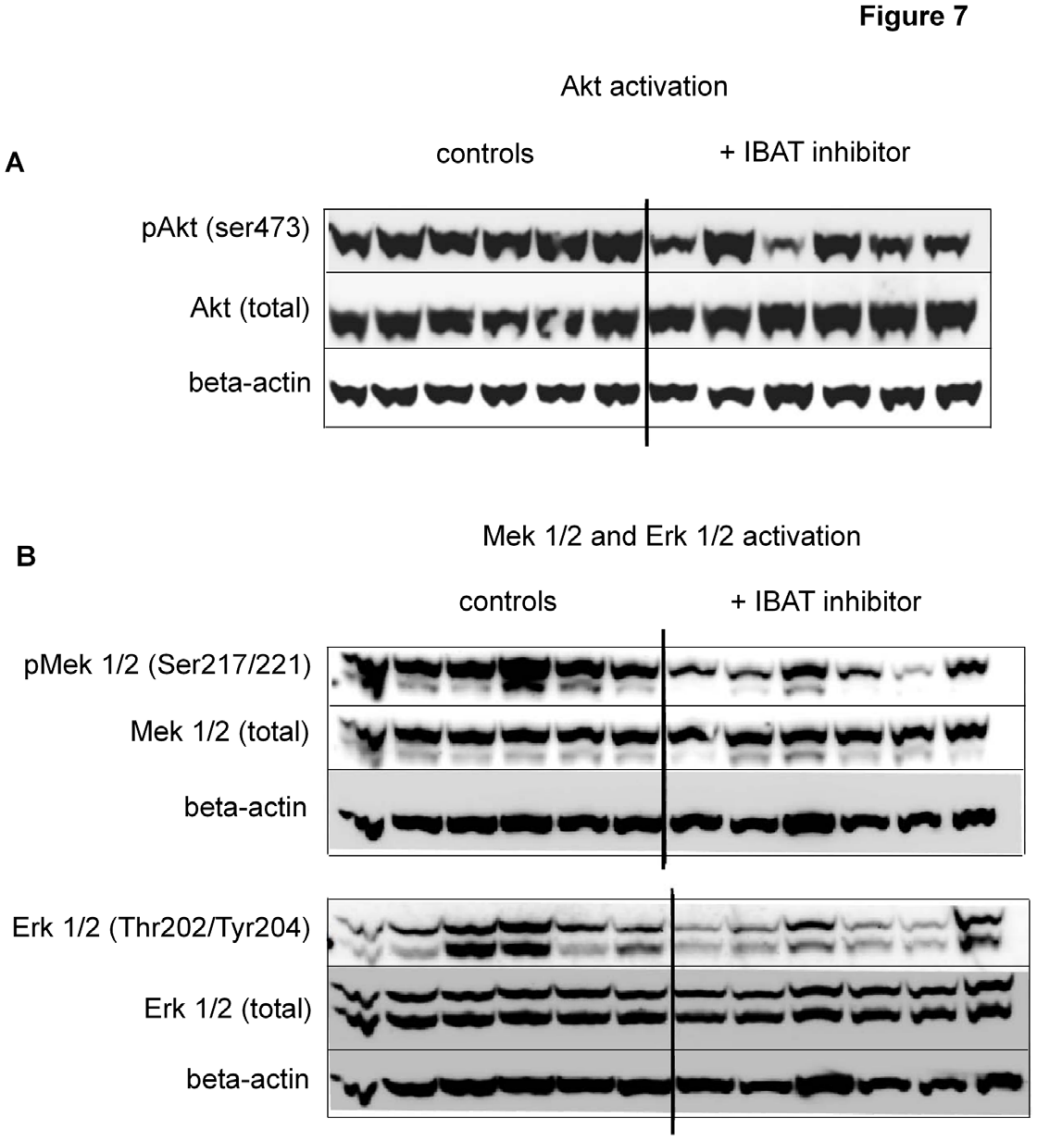
Pharmacological inhibition of Slc10a2 induces altered activity of important signal transduction pathways in ob/ob liver. The activation state of selected kinases important in glucose and lipid metabolism were investigated in individual liver protein extracts by phosphorylation site-specific antibodies in Slc10a2 inhibitor-treated and control ob/ob mice. (**A**) Western blot against liver pAkt (ser 473), the same membrane was stripped and reprobed with an antibody against total amount of Akt, (**B**) western blot against liver pMek1/2 (ser 217/221) and pErk1/2 (Thr 202/Tyr 204), the same membranes were consecutively stripped and reprobed with antibodies against total Mek1/2 and Erk1/2. All membranes were finally reprobed with an antibody against beta-actin. Blots are representative of four individual runs.

To further study the molecular mechanisms underlying the observed changes in hepatic gene transcription in the inhibitor-treated ob/ob mice, we examined the activity of major kinase signal pathways known to be important in hepatic regulation of both glucose metabolism and lipogenesis. Akt has been demonstrated to be a crucial component in regulating the hepatic response to insulin and other circulating factors with capacity to favour glycolysis and lipogenesis, and inhibit gluconeogenesis upon food intake. The induction of Srebp1c transcription by insulin is dependent on an Akt pathway [23]. When Akt activity was evaluated by phosphospecific antibodies, a reduced serine 473 phosphorylation was noted in liver lysates from inhibitor-treated animals (Figure 7A). Another kinase pathway activated by the insulin receptor and the FGFr4/beta-Klotho complex (the FGF15 receptor), is the Mek1/2– Erk1/2 pathway [24], [25]. Interestingly, we found that also these important kinases displayed reduced activation in ob/ob livers upon Slc10a2 inhibition (Figure7B).

### Inhibition of Slc10a2 and Sucrose Diet Induces Hepatic FGF21 mRNA

We have previously found that the mRNA levels for the metabolic regulator FGF21 are increased 12-fold in ob/ob livers compared to those of wt animals [26] and administration of FGF21 reduces serum TGs, insulin and glucose [27]. We here found that ob/ob mice treated with the Slc10a2 inhibitor doubled their hepatic mRNA levels of FGF21 (Figure 5C). To gain further insight in FGF21 regulation we determined FGF21 expression also in the previous sucrose feeding experiment. The basal FGF21 mRNA levels were increased 4-fold in *Slc10a2−/−* mice as compared to wt mice, and consumption of the SR diet increased FGF21 mRNA levels strongly in both wt and *Slc10a2−/−* mice (Figure 3F).

## Discussion

While describing novel findings on lipid and carbohydrate metabolism in *Slc10a2−/−* mice, this study also extends previous observations made in this animal model. Slc10a2 disruption increases the faecal loss of BAs that in turn strongly induces BA synthesis [2]. In line with this, Cyp7a1 mRNA levels were increased 7.5-fold as was the protein mass of Cyp7a1. The enzymatic activity of the Cyp7a1 protein was also induced but the level of induction was merely 2.5-fold. Together with the C4 data this suggests that substrate availability for BA synthesis may be limited in *Slc10a2−/−* mice having a major chronic loss of BAs.

Plasma cholesterol was unaltered in *Slc10a2−/−* animals. Interestingly, plasma TGs showed a significant gene-dose dependent 35% reduction in *Slc10a2−/−* mice as compared to wt controls. Further, the gene expression of four enzymes important in fatty acid synthesis (Acl, Acc, Fas and Scd1) was uniformly reduced indicating a suppressed hepatic synthesis of TG. In line with this, total TGs in the livers of *Slc10a2−/−* mice tended to be lowered, although not reaching statistical significance. The emerging pattern of a generally reduced hepatic synthesis of TGs was further supported by the fact that hepatic Srebp1c also showed a gene dose-dependent reduction in Slc10a2-deficient mice both at mRNA and protein levels.

In contrast to the situation for Srebp1c, the levels of Srebp2 mRNA were increased in *Slc10a2−/−* mice, presumably as a response to an increased demand for cholesterol. In line with this, HMG-CoA synthase and HMG-CoA reductase mRNA levels - and enzymatic activity of the latter - were all increased in *Slc10a2−/−* mice. Further evidence for an induced cholesterol deficiency in the hepatocytes was the finding of reduced gene expression for Abcg5 and Abcg8. These cholesterol transporters are induced by oxysterols via the nuclear receptor Lxrα [28]. Our finding of a discordant regulation of Srebp1c and Srebp2 mRNA is not unique, and has previously been reported following treatment of rats with a combination of a BA binding resin and mevinolin [29], [30]. Notably, there was no increase in the gene expression of the LDL receptor, which is known to show a more sluggish regulatory response compared to that of HMG-CoA reductase in mice [31].

Thus, in *Slc10a2−/−* animals, having a primary loss of BAs, regulatory responses were found not only in the pathway for cholesterol synthesis but also in pathways for TG synthesis. Important structures in carbohydrate metabolism were also altered in *Slc10a2−/−* mice. The mRNA levels of Gk, Lpk and Me were all lower in *Slc10a2−/−* mice on chow as compared to wt animals, whereas the message levels of G6pdh were increased. This concerted response should preserve the availability of plasma glucose and may result in a reduced formation of acetyl-CoA, a mutual substrate for fatty acid and isoprenoid synthesis. The metabolic demand for acetyl-CoA, utilized in isoprenoid synthesis, should be increased in *Slc10a2−/−* animals mirrored by the 3-fold increase in cholesterol synthesis in these animals (Figure 2D).

Animals were then challenged with a SR diet to unmask metabolic differences between wt and *Slc10a2−/−* mice related to lipogenesis. It was then found that *Slc10a2−/−* mice had lower levels of liver cholesterol and TG than wt mice on the SR diet. Strikingly, the levels of Srebp1c were retained at low levels even under conditions of excess substrate availability for lipogenesis, suggesting that powerful regulatory factors acting upstream of Srebp1c are specifically targeted when Slc10a2 function is abrogated. Thus, as judged from the increase in liver TG and cholesterol, as well as from the level of TG synthesis in the livers, *Slc10a2−/−* mice were more resistant to the SR diet than controls (Figures 3A and 3B).

However, following the SR-diet, the induced BA and cholesterol syntheses present in *Slc10a2*−/− mice were both reduced to levels seen in wt animals following the SR-diet. Thus, the higher resistance to the SR-diet in *Slc10a2*−/− animals could not simply be ascribed to the increased cholesterol and BA synthesis seen in these animals on regular chow. The reason for why BA synthesis was suppressed by the SR-diet is unclear, although we found that the intestinal gene expression of FGF15 was increased by 10-fold in *Slc10a2*−/− mice reaching the same level as in wt mice on this diet (Figure 4E). Given that intestinal FGF15 strongly inhibits hepatic BA synthesis after binding to FGFR4 in the liver [32] this may be one important reason for why the hepatic synthesis of BAs was lowered. However, the mechanism by which a SR-diet induces the gene expression for FGF15 in the intestine is unclear. Since these animals lack functional Slc10a2– which in the mouse appears to be the only major mechanism of active BA uptake from the intestine [2] it seems more likely that other factors than BAs may explain this remarkably strong stimulation of intestinal FGF15. Clearly, further studies are needed to understand this unexpected response.

To further explore our findings on altered lipogenesis in the Slc10a2*−/−* mice, we investigated the effects of Slc10a2 inhibition in ob/ob mice. This model of leptin deficiency is associated with peripheral insulin resistance, massive obesity and fatty liver, but may not fully represent a type 2 diabetic state. We previously showed that ob/ob mice display a suppressed BA synthesis [16]. As expected, these mice responded to Slc10a2 inhibitor administration with elevated Cyp7a1 (Figure 5C) and HMG-CoA reductase mRNA levels (not shown) while the serum levels of insulin, TG and blood glucose were all markedly reduced (Figures 5A and 4B).

Recently, studies utilizing BA binding resins have shown reductions in blood glucose and serum TG levels in ob/ob animals, and also in human diabetic subjects [33], [34]. Together with the present results, this implies that an elevated production and faecal excretion of BAs would contribute to reduce serum insulin and blood glucose levels when pathologically high. However, there are fundamental differences between the use of BA binding resins and disruption of Slc10a2 function with respect to the resulting BA pool size and composition. Specific elimination of Slc10a2 or blocking of Slc10a2-mediated BA uptake will lead to a an increased faecal loss of BAs and a reduced BA pool [2]. However, the thereby increased input of primary BAs into the large intestine will increase the formation of secondary, lipophilic BAs such as deoxycholic acid (DCA) capable of efficient feed-back inhibition of BA synthesis. This should somewhat lower the BA synthesis response and impede the restoration of the BA pool. BA binding resins on the other hand, at least in the human situation, render the resin-bound BAs unavailable to bacterial degradation into secondary BAs by resident microorganisms within the intestine. Therefore, both the levels and the relative proportion of secondary BAs such as DCA are reduced following resin treatment, which in turn will promote the stimulation of BA synthesis [35], [36] that will restore or even expand the BA pool [36]. However, it must be remembered that there are several major species differences as regards BA synthesis and its regulation [37].

In a recent study where a BA-binding resin was given to lean and db/db mice, it was found that the reduction of blood glucose and serum TGs was accompanied by an increased hepatic lipogenesis actually worsening the liver fat accumulation in these animals [36]. This response was concomitant with increased levels of hepatic Srebp1c and its target genes [36]. This adverse response of the db/db mice was shown to relate to intact Lxr and Fxr function, since Lxr- and Fxr-deficient mice given the resin did not respond with elevated Srebp1c levels. In the present study, we demonstrated that ablation of Slc10a2 instead resulted in reduced levels of both Srebp1c mRNA and mature protein which likely contributed to the resistance of lipogenesis in response to the SR diet in this model (Figure 3). Interestingly, this effect on Srebp1c was also evident when Slc10a2 was pharmacologically blocked in ob/ob mice (Figure 6A). Accordingly, the hepatic TG content was not increased under these conditions (Figure 5D). This denotes a major mechanistic difference between BA-binding resins and Slc10a2 inhibitors in mice at the diabetic state, which has previously not been demonstrated.

To seek further mechanistic explanation to the blunted induction of lipogenesis, we investigated the PI3K and Akt signalling pathway, known to be involved in Srebp1c induction by insulin. [23]. We found that the important signalling intermediate Akt displays a reduced hepatic activation in ob/ob animals treated with the inhibitor (Figure 6A). It is well known that, despite the reduced capacity of the insulin receptor to transmit signals in the liver of ob/ob mice, some downstream signal targets, including Srebp1c, are still overactive and may drive the formation of lipids in the liver [38]. This finding may explain at least part of the observed reduction of Srebp1c. Whereas Akt1 is ubiquitously expressed, Akt2 is predominantly found in the liver and other insulin responsive organs. Interestingly, Akt2 seems to have overlapping functions with Akt1 with regards to metabolism, and in a recent report describing liver-specific deletion of Akt2 in ob/ob mice it was found that these animals have reduced hepatic production of lipids with lowered mRNA levels of Srebp1c and its target genes Fas and Acc [39]. Since the antibodies employed to detect the ser473-phosphorylated and total Akt are known to recognize both Akt1 and Akt2, this assay cannot distinguish what variants that are affected by the inhibitor treatment (Figure 6A).

Another pathway utilized by several cell surface receptors, including receptor tyrosine kinases like the insulin receptor, is the classical Mek1/2– Erk1/2 pathway [40]. We found this main pathway to be suppressed in the liver under conditions of Slc10a2 inhibition. This reduction was also observed in the *Slc10a2+/−* and *Slc10a2−/−* livers in a dose dependent manner (not shown). Interestingly, this pathway is also shared with the FGFr4 and its co-receptor beta-Klotho, serving as an FGF15 receptor complex [25]. Thus, a reduction in activity of this pathway may be a prerequisite for the induction of BA production via Cyp7a1. It is in this respect also of interest to note that when mice deficient in FGFr4 are challenged with a high fat diet, they respond with lower hepatic TG production as compared to wt controls [24]. In this sense, abrogation of Slc10a2, with dramatically reduced levels of the FGFr4 ligand, FGF15, would likely mimic this situation with respect to induction of hepatic lipogenesis upon dietary challenge.

FGF15 and FGF19 have been shown to have effects on energy metabolism. Overexpression or injection of FGF19 into mice has been shown to affect metabolic rate and improves insulin resistance and dyslipidemia. It must be noted that these studies have rendered FGF15/FGF19 plasma levels several powers of magnitude higher than those observed at normal conditions in a healthy human/animal. In a previous study by Moschetta and colleagues [41] it was shown that Slc10a2−/− mice have very low levels of FGF15 mRNA in the distal part of the small intestine. We could confirm this finding in the current report. It is therefore unlikely that FGF15 contributes to the positive antidiabetic effects observed in this study when an Slc10a2 inhibitor was administrated to ob/ob animals. Instead, we demonstrate that in accordance with studies using BA binding resins, FGF15 is not essential for improved plasma lipid or glucose levels.

The recently discovered metabolic regulator FGF21 was doubled in ob/ob mice receiving the Asbt inhibitor (Figure 5C) and was increased by 4-fold in *Slc10a2−/−* mice (Figure 3F). Since treatment of ob/ob or db/db mice with high doses of FGF21 reduces elevated blood sugar and TGs [27] it may be possible that FGF21 is involved in the reduction of blood glucose and TGs in ob/ob mice and in the reduction of TGs in *Slc10a2−/−* mice. However, the facts that FGF21 is already increased over 10-fold in ob/ob mice [26] and that FGF21 was strongly increased up to 25-fold upon challenge with the SR diet to wt or to *Slc10a2−/−* mice do not support such a possibility. An alternative explanation may be that *Slc10a2−/−* and ob/ob mice have at the basal level an increased level of metabolic stress as can also be induced in animals challenged with high doses of dietary sucrose or an Asbt inhibitor. It is known that mice respond with increased hepatic expression of FGF21 when subjected to fasting [42] or to high energy diets [43]. Thus, the limited 1-fold stimulation of hepatic FGF21 mRNA during treatment with an Asbt inhibitor does not appear a likely explanation for why plasma glucose is reduced.

Recently, the nuclear receptors RAR-related orphan receptor (Ror)α and Rorγ were shown to bind 7alpha-hydroxycholesterol with high affinity [44]. Binding of these compounds was demonstrated to repress the transcription of both Pepck and G6Pase in a Ror-dependent manner. It was suggested that an increased activity of Cyp7a1 would repress the hepatic glucose output via increased availability of ligands for these Rors [44]. It may be speculated that the decrease in Pepck and G6Pase observed in our study could in part also relate to an increased gene-specific repression exerted by these nuclear receptors.

In summary, this work demonstrates that abrogation of the ileal BA transporter Slc10a2 leads to reduced serum TG levels with concomitantly lowered hepatic Srebp1c levels. This feature is further pronounced when Slc10a2 animals are challenged with a lipogenic diet, manifested by a suppressed hepatic lipogenesis. Our work also reveals novel mechanistic differences between the use of BA-binding resins and specific inhibition of the ileal BA transporter in the diabetic ob/ob model. Inhibition of Slc10a2 appears to be a more favourable option in treating elevated serum TG and blood glucose levels, since it does not exert the adverse effects on lipogenesis seen with BA binding resins.

Further studies are needed to define the global hormonal response including intestinal incretins, and the precise pathways affected in the liver under conditions of Slc10a2 functional abrogation in the diabetic state, and could lead to novel therapeutic interventions in hyperlipidemic states of disease, including type 2 diabetes.

## Supporting Information

Figure S1 **(A)** Schematic overview of vector and strategy used to target the *Slc10a2* wt allele in order to obtain a *Slc10a2* null mouse. **(B)** A representative immunoblot employing a specific antibody directed against the Slc10a2 protein demonstrates absence of Slc10a2 protein expression in ileum of *Slc10a2−/−* mice.
(TIF)

Text S1 **Targeting the** ***Slc10a2*** **locus.**
(DOC)
