# Supplementary figures and images for: Inhibition of Intestinal Bile Acid Transporter Slc10a2 Improves Triglyceride Metabolism and Normalizes Elevated Plasma Glucose Levels in Mice
Source: PLoS One. 2012 May 25;7(5):e37787. doi: 10.1371/journal.pone.0037787 (PMC3360597; doi:10.1371/journal.pone.0037787)

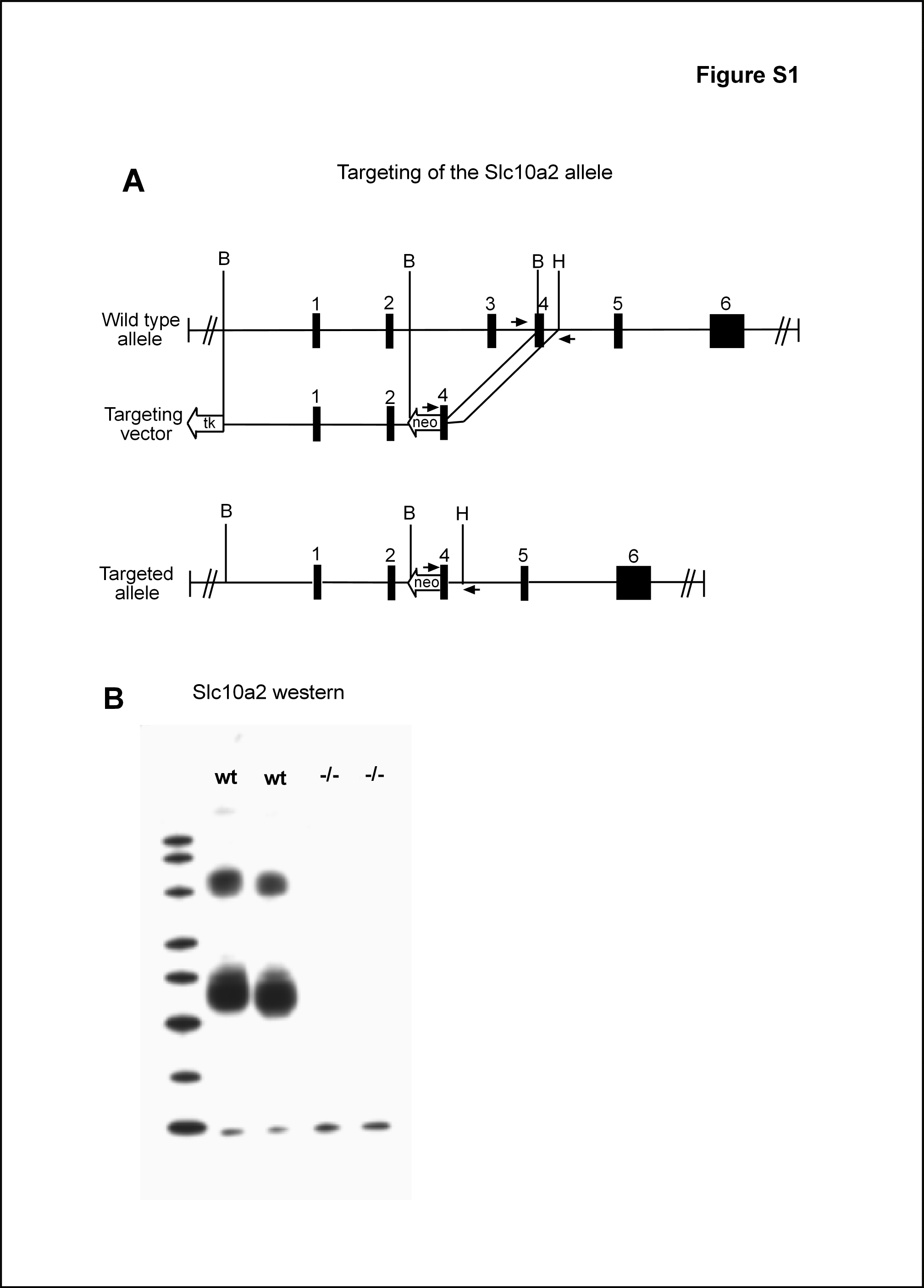

Supplement: Figure S1 — (A) Schematic overview of vector and strategy used to target the Slc10a2 wt allele in order to obtain a Slc10a2 null mouse. (B) A representative immunoblot employing a specific antibody directed against the Slc10a2 protein demonstrates absence of Slc10a2 protein expression in ileum of Slc10a2−/− mice. (TIF) [file pone.0037787.s001.tif]
