# Supplementary material for: Inhibition of Intestinal Bile Acid Transporter Slc10a2 Improves Triglyceride Metabolism and Normalizes Elevated Plasma Glucose Levels in Mice
Source: PLoS One. 2012 May 25;7(5):e37787. doi: 10.1371/journal.pone.0037787 (PMC3360597; doi:10.1371/journal.pone.0037787)
Supplement: Text S1 — Targeting the Slc10a2 locus. (DOC) [file pone.0037787.s002.doc]

**Supporting information**

**Supplemental experimental procedures**

**Targeting the *Slc10a2* locus**

The targeting vector used to modify the mouse *Slc10a2* locus was a kind gift from P. Dawson and has been previously described (2). In brief, it consisted of a ~14kb 5’ homology arm, an inverted Neo (neomycin phosphotransferase) cassette driven off the PGK (phosphoglycerate kinase) promoter and a 1.6kb 3’ homology arm. The targeting vector was designed so that correct targeting would result in most of intron 2, exon 3, intron 3 and the very 5’ end of exon 4 being deleted and replaced by the Neo cassette in order to inactivate the *Slc10a2* gene (see **Figure S1A**). *B, BamHI; H, HaeIII.*

After linearization, the targeting construct was electroporated into R1 ES cells (derived from 129/SvJ) and neomycin-resistant clones were selected in G-418-containing (300 μg/ml) media. Of 400 G418-resistant clones screened, 2 targeted clones were identified using a PCR screening over the short arm and then confirmed by Southern analysis. The primers used for detecting the targeted allele were a forward primer located in the inverted Neo cassette and a reverse primer located downstream of the short arm (5´-cgtactggggcatagaatctttgc-3´). The same reverse primer was combined with a forward primer in intron 3 (5´-ctcttcctatgaagctaaaggggc-3´) for detection of the wild-type allele. One positive clone was expanded and injected into C57Bl/6 blastocysts to generate chimeric mice. Chimeric males were backcrossed to C57Bl/6 females in at least 8 generations and genotyping of the offspring was performed from tail biopsies by both PCR and Southern to confirm germ-line transmission.

To verify that the targeted Slc10a2 allele resulted in a null mutation, total RNA was prepared from the kidneys and intestines of 8-10 week old homozygous, heterozygous, and wild-type littermates using TRIzol Reagent according to the manufacturer´s instructions (Invitrogen, Paisley, UK). cDNA was synthezised using Super-script^TM^ II Rnase H^-^ Reverse Transcriptase and random hexamer primers (Invitrogen, Paisley, UK). TaqMan real-time PCR was performed using the ABI PRISM 7700 Sequence Detector System (Applied Biosystems, Warrington, UK). All samples were run in triplicate and data were normalized using the mouse acidic ribosomal phosphoprotein PO (M36B4) as an internal control. The TaqMan primers and probe for Slc10a2 were: 5´-accacttgctccacactgctt-3´ (forward), 5´-acccacatcttggtgtagacga-3´ (reverse) and 5´-ccttggaatgatgcctctttgcctc-3´ (probe).
